# Use of three-dimensional technology in tracheal intubation: an alternative to minimize the contamination risk by COVID-19

**DOI:** 10.31744/einstein_journal/2020CE6118

**Published:** 2020-11-05

**Authors:** Maria Carolina Barbosa Teixeira Lopes, Luca Silveira Bernardo, Eduardo Sartori de Camargo, Graciana Maria de Moraes, Luiz Humberto Vieri Piacezzi, Ruth Ester Assayag Batista, Cássia Regina Vancini-Campanharo

**Affiliations:** 1 Universidade Federal de São Paulo Escola Paulista de Enfermagem São PauloSP Brazil Escola Paulista de Enfermagem, Universidade Federal de São Paulo, São Paulo, SP, Brazil.; 2 Universidade Federal de São Paulo Escola Paulista de Medicina São PauloSP Brazil Escola Paulista de Medicina, Universidade Federal de São Paulo, São Paulo, SP, Brazil.; 3 Universidade de São Paulo São CarlosSP Brazil Universidade de São Paulo, São Carlos, SP, Brazil.

Dear Editor,

Because of the spreading of acute respiratory syndrome coronavirus (SARS-CoV-2) the use of personal protective equipment was adopted by whole-population and, within a short period of time, these medical supplies became scarce and expensive, therefore leading to a severe global crisis, mainly in health services. Based on this scenario, rapid and low-cost methods are required to stop the virus spread.^(^^1^^,^^2^^)^

Three-dimensional printing has enabled the production of equipment such as videolaryngoscope systems in a timely fashion and with low cost.^(^^2^^)^ Videolaryngoscopy makes tracheal intubation more successful.^(^^3^^)^ In addition, in COVID-19 cases this equipment may increase the distance between the providers and the patients airways, facilitate the interventions of health care professional, reduce duration of procedure, and minimize the contamination.^(^^3^^-^^5^^)^

In cases of the collapse of the health resources, such in COVID-19 pandemic, the three-dimensional printing technology may be an efficient and low-cost alternative, particularly in countries with limited resources.
