# Supplementary material for: Use of three-dimensional technology in tracheal intubation: an alternative to minimize the contamination risk by COVID-19
Source: Einstein (Sao Paulo). 2020 Nov 5;18:eCE6118. doi: 10.31744/einstein_journal/2020CE6118 (PMC7647383; doi:10.31744/einstein_journal/2020CE6118)
Supplement: Translation

# Translation: Uso da tecnologia tridimensional na intubação traqueal: uma alternativa para minimizar o risco de contaminação por COVID-19

**Authors:** Maria Carolina Barbosa Teixeira Lopes, Luca Silveira Bernardo, Eduardo Sartori de Camargo, Graciana Maria de Moraes, Luiz Humberto Vieri Piacezzi, Ruth Ester Assayag Batista, Cássia Regina Vancini-Campanharo

**Affiliations:** 1 São PauloSP Brasil Escola Paulista de Enfermagem, Universidade Federal de São Paulo, São Paulo, SP, Brasil.; 2 São PauloSP Brasil Escola Paulista de Medicina, Universidade Federal de São Paulo, São Paulo, SP, Brasil.; 3 São CarlosSP Brasil Universidade de São Paulo, São Carlos, SP, Brasil.

Caro Editor,

Devido à disseminação do coronavírus da síndrome respiratória aguda grave 2 (SARS-CoV-2), a utilização de equipamentos de proteção foi adotada pela população e, em pouco tempo, eles se tornaram escassos e onerosos, ocasionando grave crise global, principalmente nos serviços de saúde. Diante desse cenário, soluções rápidas e de menor custo tornaram-se necessárias para conter a propagação do vírus.^(^^1^^,^^2^^)^

A impressão tridimensional tem possibilitado a confecção de equipamentos, como sistemas de videolaringoscopia, em tempo hábil e com baixo custo.^(^^2^^)^ A videolaringoscopia torna a intubação traqueal mais assertiva.^(^^3^^)^ Além disso, nos casos de COVID-19, aumenta a distância entre o rosto do profissional e a via aérea do paciente, facilita as intervenções do profissional assistente e pode diminuir a duração do procedimento, reduzindo a chance de contaminação.^(^^3^^-^^5^^)^

Em casos de colapso dos recursos em saúde, como na pandemia da COVID-19, a impressão de equipamentos, por tecnologia tridimensional, pode ser uma alternativa eficaz e de baixo custo, sobretudo em locais com poucos recursos.
